# Impact of Breakfasts (with or without Eggs) on Body Weight Regulation and Blood Lipids in University Students over a 14-Week Semester

**DOI:** 10.3390/nu5125097

**Published:** 2013-12-16

**Authors:** Janice M. Rueda, Pramod Khosla

**Affiliations:** Department of Nutrition and Food Science, Wayne State University, Detroit, MI 48202, USA; E-Mail: rueda@wayne.edu

**Keywords:** college freshmen, breakfast, body weight, plasma lipids, eggs, dietary cholesterol

## Abstract

The effects of breakfast type on body weight and blood lipids were evaluated in university freshman. Seventy-three subjects were instructed to consume a breakfast with eggs (Egg Breakfast, EB, *n* = 39) or without (Non-Egg Breakfast, NEB, *n* = 34), five times/week for 14 weeks. Breakfast composition, anthropometric measurements and blood lipids were measured at multiple times. During the study, mean weight change was 1.6 ± 5.3 lbs (0.73 ± 2.41 kg), but there was no difference between groups. Both groups consumed similar calories for breakfast at all time-points. The EB group consumed significantly more calories at breakfast from protein, total fat and saturated fat, but significantly fewer calories from carbohydrate at every time-point. Cholesterol consumption at breakfast in the EB group was significantly higher than the NEB group at all time points. Breakfast food choices (other than eggs) were similar between groups. Blood lipids were similar between groups at all time points, indicating that the additional 400 mg/day of dietary cholesterol did not negatively impact blood lipids.

## 1. Introduction

It has been estimated that 35% of college students in the United States could be overweight or obese, putting them at increased risk for cardiovascular disease (CVD) [1,2]. Therefore preventing obesity and associated metabolic dysfunctions in young adults represents an opportunity to reduce the growing public health burden of CVD [3,4,5,6,7]. Regular breakfast consumption is a common behavioral characteristic of weight maintenance and is also associated with reduced risk for CVD [8]. Compared to those who skipped breakfast, subjects who consumed breakfast had lower total cholesterol (TC) and low-density lipoprotein (LDL) cholesterol, as well as greater insulin sensitivity [9]. The impact of breakfast consumption on attenuation of weight gain among college students has not been assessed.

Consumption of higher satiety foods is associated with decreased food intake at subsequent meals, as well as reduced intake in the short term. In a study measuring food consumption 2 h following a test meal, an inverse relationship was shown between meal satiety score and kilocalories consumed [10]. Foods with higher protein, fiber and water content have greater effects on satiety than foods higher in fat and carbohydrates. When comparing high fat, high protein or high carbohydrate breakfasts and subsequent energy intake, the high-fat breakfast resulted in greater subjective hunger before lunch compared to the protein or carbohydrate breakfasts [11].

Egg consumption is associated with increased satiety and reduced overall caloric intake. Overweight and obese subjects who consumed an egg breakfast reported significantly greater feelings of satiety through 90 min after the meal and consumed less overall energy than subjects fed an isocaloric bagel breakfast [12]. An eight-week study of overweight men and women on reduced calorie diets showed that those consuming egg breakfasts had a 65% greater average reduction in weight compared to those assigned to bagel breakfasts [13].

With the association of high levels of TC and LDL-C with increased risk of CVD, reducing dietary cholesterol became a highly advocated strategy to reduce that risk. Since eggs contribute approximately 30% of the dietary cholesterol to the diets of Americans [14], they were discouraged or limited from weight-loss plans. However, recent epidemiological evidence has shown no association between egg consumption (one egg per day) and CVD risk, although increased risk was seen among diabetic subjects consuming more than one egg per day [15]. Consumption of 2 eggs per day for 6 weeks had no effect on TC or LDL cholesterol [16]. An intervention with dietary cholesterol intakes of 640 mg per day actually resulted in an increase in the less atherogenic large LDL particles among those classified as “hyper-responders” to dietary cholesterol [17].

The current study, evaluated the role of different types of breakfast (with and without eggs) on weight management and plasma lipids in a sample of students during the fourteen weeks of their first semester in college.

## 2. Experimental Section

**Figure 1:**
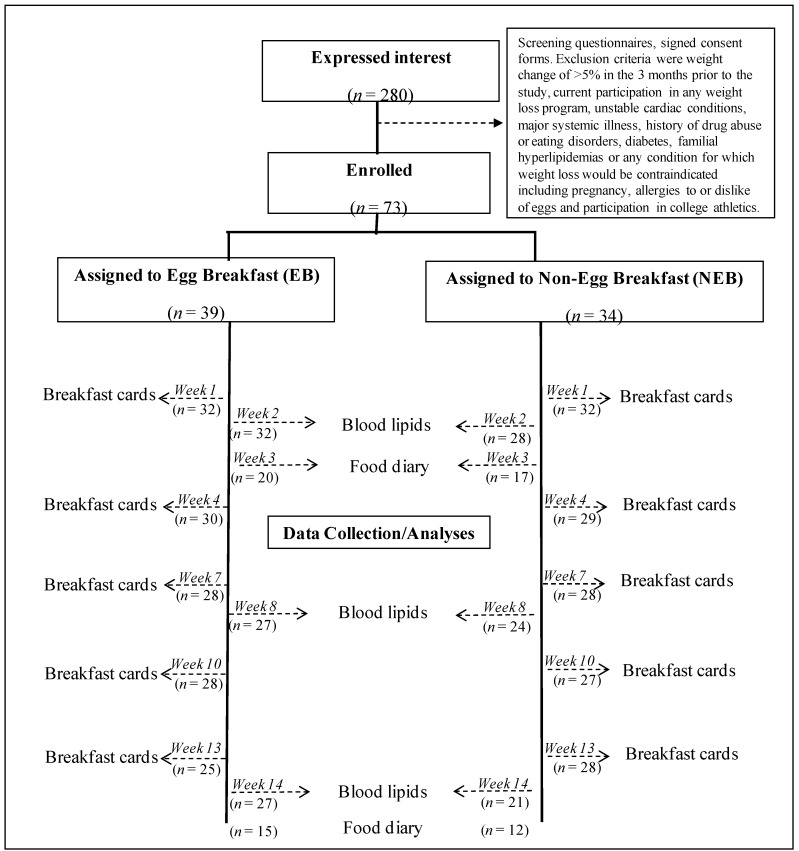
Study design.

The study was carried out in the Fall semester of 2009. Subjects were freshmen university students, aged 17–20, living in the residence halls and enrolled in the university meal plan. Subjects were recruited by both passive (response to study advertisements) and active methods (directly by a group of research assistants trained by the lead researcher to provide information about the study). This phase occurred during the move-in period but before the start of classes. In all, 280 students indicated interest in the study. Actual enrollment for the study was conducted over a period of three days during the first week of the semester. A total of 73 subjects were enrolled (Figure 1). Participants were compensated for the breakfast portion of their pre-paid meal plans, in proportion to each student’s actual participation in the study. The study protocol was approved by the Human Investigation Committee of Wayne State University (HIC #: 032508MP2E, Protocol #: 0906007205) and informed written consent was obtained from each subject.

Subjects were asked to consume an Egg-Based Breakfast or a Non-Egg Based Breakfast (5 days a week) for 14 weeks. Blood lipid analyses, 3-day food diaries and breakfast cards were collected at the times indicated. The number of subjects from which data was obtained at each time point is also indicated. Please refer to text for further details.

Study participants were randomly assigned to either an Egg Breakfast (EB) or a Non-Egg Breakfast (NEB), to be consumed in the residence hall dining facility 5 days per week, for the duration of the 14-week study. For at least 5 days each week, EB subjects were required to include one “serving” of eggs (equivalent to 2 whole eggs), while NEB subjects were instructed to exclude eggs. All study breakfasts were otherwise *ad libitum* and were consumed in the university dining facility during designated breakfast service times.

Compliance was monitored two ways. Frequency of breakfast consumption was assessed from participants’ university-issued identification cards, which are required for entry into residence hall dining facilities. Secondly, compliance to breakfast group instruction was assessed by immediate written recall of breakfast items consumed. Subjects were asked to fill out “breakfast cards” (at weeks 1, 4, 7, 10, and 13) immediately upon exiting the dining hall, in the presence of research assistants. At each time point, breakfast cards were collected for 3 days (Tuesday, Wednesday and Thursday mornings), to obtain an average snapshot of consumption for each subject, and to allow for the possibility that subjects were not able to attend breakfast on a particular day. The purpose of the “breakfast cards” was to assess compliance to study group assignment as well as to provide an assessment of the nutritional profile of breakfasts consumed. All available items were of a standard, predetermined serving size, and self-serve items used utensils of known volume. Macronutrient, fiber and cholesterol content were determined using the information provided by AVI Food Systems [18].

Food diaries were collected during the third week of the study (after first full week of classes) and again at the final measurement collection session during the last week of the study (last full week of classes). Study participants were verbally instructed as to how to complete their food diaries by the lead researcher, and shown examples illustrating the level of detail required. Subjects were also given written instructions. Food diaries were analyzed using an online database created and maintained by the University of Illinois Department of Food Science and Human Nutrition [19]. The database is composed of the USDA Handbook #8 [20] as well as data from various food companies.

Anthropometric measurements were taken in a residence hall activity room in the mornings prior to breakfast consumption at weeks 2, 4, 8, 11 and 14. Height and weight were measured with subjects wearing light clothing with all materials removed from pockets. Weight and body composition were assessed with a Tanita body composition analyzer (model TBF-300A, Arlington Heights, IL, USA).

Fasting plasma lipids were assessed at weeks 2, 8 and 14. Subjects reported to the blood draw sessions in a fasted state, having not consumed any food or drink after going to sleep the night before (no later than 10:00 PM was given as a guideline). Blood was drawn from seated subjects by trained phlebotomists, into 2 × 5 mL vacutainer tubes containing EDTA (Becton-Dickinson, Franklin Lakes, NJ, USA), which were kept on ice until transfer back to the laboratory.

Plasma was separated by centrifugation. Total cholesterol, HDL-cholesterol and Triglyceride (TG) were determined by enzymatic assays (Pointe Scientific, Canton, MI, USA). Blood samples were also assessed for glucose, total cholesterol, HDL cholesterol, LDL cholesterol and TG using Cholestech LDX mobile units using Lipid Profile Plus Glucose cassettes (Cholestech, Hayward, CA, USA). Fingerstick blood draws were used on subjects who either refused venous sampling or experienced unsuccessful venous blood draws. Values obtained from this portable screening system were compared to lab-based enzymatic assays performed the same day of sample collection. 

All data were analyzed with SPSS version 17.0 statistical analysis software (Chicago, IL, USA). Unless otherwise indicated, results are reported as means ± SD. Independent and repeated measures *t*-tests were used to compare means between two groups. In addition to analysis by intervention group (EB *versus* NEB), results were analyzed by gender, baseline BMI category (<25 kg/m^2^, ≥25 kg/m^2^), BMI health categories (Underweight, Healthy Weight, Overweight, Obese), final weight change categories (Lost Weight, No Weight Change, Gained Weight) and by study participation categories (Completed Final Measurement, Did Not Complete Final Measurement). Analysis of variance (ANOVA) was used to compare means between groups, and chi-square tests were used to compare proportions within categorical variables. All statistical tests were based on α ≤ 0.05 as a level of significance.

## 3. Results

Baseline characteristics of the subjects are detailed in Table 1. There was no significant difference between experimental groups for any parameter with the exception of body weight and BMI. There was no correlation between baseline weight or BMI status in differences in outcome measures at the final measurement. Fifty-seven subjects (78%) completed the final anthropometric measurements and blood draws at week 14, which is a comparable retention to similar, previously reported studies. There was no significant difference in baseline anthropometric measures, fasting plasma cholesterol or blood glucose measures between those who completed final measurement of the study and those who did not (data not shown).

**Table 1:** Baseline characteristics of subjects.

	Egg Group	Non-Egg Group	*p*
	(*n* = 39)	(*n* = 34)	
Female	23 (59%)	23 (68%)	NS ^a^
Male	16 (41%)	11 (32%)	NS
Mean Height (m)	1.73 ± 0.02	1.71 ± 0.02	0.4
Mean Weight (kg)	83.9 ± 5.1 *	70.6 ± 2.6 *	0.029
Mean BMI (kg/m^2^)	27.8 ± 1.5 *	24.1 ± 5.2 *	0.046
BMI < 25 kg/m^2^	18 (46%)	24 (71%)	0.031 ^b^
BMI ≥ 25 kg/m^2^	21 (54%)	10 (29%)	0.031
Underweight	6 (15%)	4 (12%)	NS ^c^
Healthy Weight	12 (31%)	20 (59%)	NS
Overweight	8 (21%)	3 (9%)	NS
Obese	13 (33%)	7 (21%)	NS
Mean % Fat	28.0 ± 15.4	23.7 ± 12.4	NS
Total Cholesterol (mg/dL)	145 ± 22	147 ± 27	NS
Triglycerides (mg/dL)	83 ± 36	92 ± 38	NS
LDL Cholesterol (mg/dL)	82 ± 19	83 ± 21	NS
HDL Cholesterol (mg/dL)	49 ± 12	49 ± 14	NS

Values are Means ± SD. Significant differences in means between groups were determined using independent samples *t*-tests. * *p* < 0.05. Significant differences in proportions between groups were determined using Chi-square analysis. ^a^ No significant difference in proportions of females and males were found between breakfast groups, Chi-square(1) = 0.891, *p* = 0.461. ^b^ A significant difference in proportions of subjects with baseline BMI < 25 and those with baseline BMI ≥ 25 between breakfast groups, Chi-square(1) = 5.193, *p* = 0.031. ^c^ No significant difference in proportions of subjects in BMI health categories were found between breakfast groups, Chi-square(3) = 6.799, *p* = 0.079.

**Figure 2:**
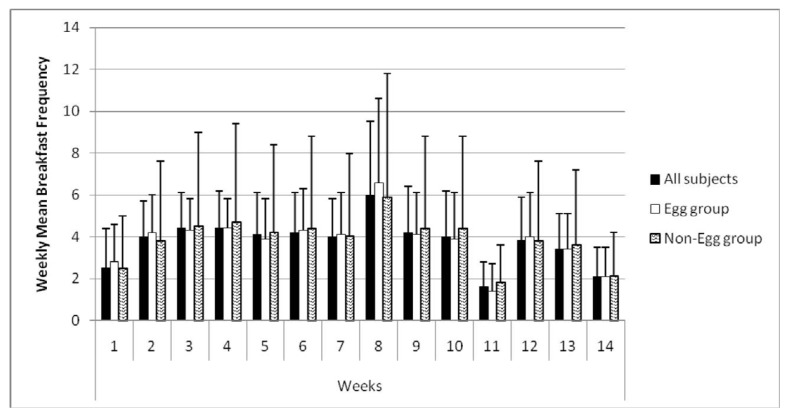
Time course of breakfast frequency.

Reported weekly means of breakfast frequency were determined by analyzing data from university issued student ID-cards. Each time students entered a dining facility, their ID card was swiped through an electronic reader, and this information was collected from all residence hall dining facilities. Data for each subject from each residence hall were combined for analysis (Figure 2). A general time effect was observed with breakfast frequency increasing from week 1 (2.5 ± 1.9) over the course of the semester (*p* < 0.0001), and no significant difference between genders was observed (*p* = 0.719). Repeated measures analysis for all subjects showed no significant mean changes from baseline in breakfast attendance frequency until week 4, where a mean increase was noted (0.5 ± 1.8, *p* = 0.031) and then again at week 8 (2.1 ± 3.4, *p* < 0.0001). No significant changes from baseline were seen again (with the exception of week 11, which corresponded to the Thanksgiving holiday break) until a significant decline in breakfast attendance frequency at week 13 (−0.5 ± 2.1, *p* = 0.038) and week 14 (−1.9 ± 2.0, *p* < 0.0001). There was no significant difference between groups in the mean frequency of breakfast attendance per week (EB = 3.7 ± 1.4, NEB = 3.8 ± 1.5, *p* = 0.731). The relatively high frequency of overall average frequency of breakfasts attended per week indicated an acceptable level of compliance to study requirements. There was no significant difference in mean weekly breakfast frequency seen between intervention groups.

Values are Mean ± SD (*n* = 73 All subjects, *n* = 39 Egg group, *n* = 34 Non-Egg group). Breakfast frequency was assessed using University-issued Student ID cards. Week 8 was Halloween—and most students stayed on campus over the weekend—hence the higher breakfast consumption.

Nutrient composition of breakfasts is shown in Table 2. Subjects completed recall cards of items and amounts-consumed during breakfast immediately upon leaving the dining hall for three days during each of the 5 measurement periods. At week 1, there were no significant differences observed between groups in calories consumed at breakfast or calories per kilogram body weight. At week 1, EB subjects showed significantly higher mean breakfast consumption of fat, saturated fat, cholesterol and protein, while NEB subjects showed higher mean carbohydrate intake at breakfast. In the EB group, one serving of scrambled eggs contained 170 calories, 12 g of fat, 3.5 g of saturated fat and 385 mg of cholesterol. Most common breakfast choices for those in the NEB group were pancakes, which had 370 calories per serving but only 8 g of fat, no saturated fat and no cholesterol, and ready to eat cereal, which had approximately 115 calories per serving one gram fat, no saturated fat and no cholesterol. The breakfast nutrient composition data thus reflected the expected differences between the EB and NEB groups. These differences were essentially maintained for the duration of the study period (with the exception of non-significant differences in calories from carbohydrates at week 7 and calories from protein at week 10). Dietary cholesterol intake—a strong measure of compliance to the breakfast treatment—was significantly higher in the Egg group at all time points.

**Table 2:** Breakfast nutrient composition (per day).

	Egg Group	Non-Egg Group	*p*
*Total Calories*			
Week 1	(*n* = 32) 615 ± 130	(*n* = 30) 591 ± 197	NS
Week 4	(*n* = 30) 680 ± 212	(*n* = 29) 633 ± 244	NS
Week 7	(*n* = 28) 686 ± 176	(*n* = 28) 611 ± 197	NS
Week 10	(*n* = 28) 633 ± 189	(*n* = 27) 679 ± 241	NS
Week 13	(*n* = 25) 601 ± 167	(*n* = 28) 641 ± 180	NS
*Calories/kg Body Weight*			
Week 1	8.3 ± 2.9	8.9 ± 3.9	NS
Week 4	9.3 ± 3.4	9.6 ± 4.9	NS
Week 7	9.3 ± 3.2	9.2 ± 3.5	NS
Week 10	8.4 ± 3.5	10.4 ± 4.7	NS
Week 13	8.4 ± 3.7	9.7 ± 3.6	NS
*Calories from Carbohydrates*			
Week 1	301 ± 96	402 ± 139	0.002
Week 4	325 ± 154	418 ± 144	0.002
Week 7	335 ± 125	400 ± 134	NS
Week 10	284 ± 113	440 ± 129	<0.0001
Week 13	265 ± 106	427 ± 129	<0.0001
*Calories from Protein*			
Week 1	95 ± 28	69 ± 30	0.001
Week 4	111 ± 37	75 ± 36	<0.0001
Week 7	106 ± 27	76 ± 34	0.001
Week 10	102 ± 38	84 ± 34	NS
Week 13	97 ± 30	77 ± 29	0.014
*Calories from Total Fat*			
Week 1	217 ± 59	127 ± 64	<0.0001
Week 4	241 ± 75	127 ± 64	<0.0001
Week 7	243 ± 63	142 ± 64	<0.0001
Week 10	243 ± 83	163 ± 91	0.001
Week 13	235 ± 68	144 ± 59	<0.0001
*Calories from Saturated Fat*			
Week 1	56 ± 17	32 ± 16	<0.0001
Week 4	60 ± 23	33 ± 16	<0.0001
Week 7	61 ± 17	36 ± 18	<0.0001
Week 10	60 ± 22	35 ± 18	<0.0001
Week 13	60 ± 16	33 ± 15	<0.0001
*Cholesterol (mg)*			
Week 1	414 ± 41	63 ± 96	<0.0001
Week 4	422 ± 27	46 ± 81	<0.0001
Week 7	418 ± 14	76 ± 119	<0.0001
Week 10	417 ± 18	49 ± 79	<0.0001
Week 13	404 ± 52	68 ± 114	<0.0001

Breakfast cards were collected at weeks 1, 4, 7, 10 and 13 on three consecutive days. For each subject the average daily nutrient intake over this 3-day period was calculated. Values are Mean ± SD. NS, not significant.

To address whether those who consumed eggs for breakfast were also more likely to choose other items compared to the NEB group, frequencies of consumption of specific breakfast items were analyzed from the breakfast card data. Therefore mean daily frequencies of the consumption of specific breakfast items were determined for each breakfast card collection time point (Eggs, Ready-to-Eat Cereals, Hot Cereals, Pancakes, Meats, Potatoes, Toast, Pastries, Juice, Milk, Fruit, Yogurt) and are shown in Table 3. This was calculated for each subject as the sum of the number of servings of each item on each day a breakfast card was completed, divided by the number of breakfast occasions (defined as the number of breakfast cards the subject turned in for a particular week).

**Table 3:** Mean daily frequency of breakfast item servings consumed.

	Egg Group	Non-Egg Group	*p*
*Eggs*			
Week 1	(*n* = 32) 0.98 ± 0.08	(*n* = 30) 0.56 ± 0.21	<0.0001
Week 4	(*n* = 30) 1.00 ± 0.06	(*n* = 29) 0.03 ± 0.18	<0.0001
Week 7	(*n* = 28) 1.00 ± 0.00	(*n* = 28) 0.07 ± 0.26	<0.0001
Week 10	(*n* = 28) 0.95 ± 0.20	(*n* = 27) 0.04 ± 0.19	<0.0001
Week 13	(*n* = 25) 0.97 ± 0.12	(*n* = 28) 0.07 ± 0.26	<0.0001
*Ready-to-Eat Cereal*			
Week 1	0.21 ± 0.33	0.24 ± 0.32	NS
Week 4	0.23 ± 0.40	0.34 ± 0.42	NS
Week 7	0.21 ± 0.36	0.26 ± 0.35	NS
Week 10	0.09 ± 0.27	0.38 ± 0.41	0.004
Week 13	0.15 ± 0.00	0.30 ± 0.38	NS
*Hot Cereal*			
Week 1	0.06 ± 0.21	0.13 ± 0.29	NS
Week4	0.12 ± 0.31	0.14 ± 0.33	NS
Week 7	0.10 ± 0.24	0.10 ± 0.24	NS
Week 10	0.11 ± 0.29	0.07 ± 0.27	NS
Week 13	0.06 ± 0.22	0.10 ± 0.28	NS
*Pancakes*			
Week 1	0.21 ± 0.34	0.38 ± 0.48	NS
Week 4	0.38 ± 0.49	0.45 ± 0.45	NS
Week 7	0.21 ± 0.33	0.40 ± 0.46	NS
Week 10	0.30 ± 0.40	0.51 ± 0.43	NS
Week 13	0.15 ± 0.31	0.48 ± 0.47	0.005
*Breakfast Meats*			
Week 1	0.41 ± 0.40	0.36 ± 0.44	NS
Week 4	0.49 ± 0.54	0.36 ± 0.47	NS
Week 7	0.51 ± 0.46	0.42 ± 0.41	NS
Week 10	0.53 ± 0.50	0.40 ± 0.53	NS
Week 13	0.50 ± 0.44	0.36 ± 0.41	NS
*Potatoes*			
Week 1	0.40 ± 0.43	0.38 ± 0.45	NS
Week 4	0.37 ± 0.42	0.49 ± 0.48	NS
Week 7	0.51 ± 0.45	0.41 ± 0.45	NS
Week 10	0.54 ± 0.43	0.59 ± 0.49	NS
Week 13	0.57 ± 0.47	0.45 ± 0.45	NS
*Toast*			
Week 1	0.14 ± 0.28	0.28 ± 0.38	NS
Week 4	0.12 ± 0.24	0.21 ± 0.33	NS
Week 7	0.19 ± 0.36	0.31 ± 0.38	NS
Week 10	0.07 ± 0.21	0.26 ± 0.40	0.031
Week 13	0.14 ± 0.31	0.27 ± 0.39	NS
Pastries			
Week 1	0.08 ± 0.17	0.18 ± 0.34	NS
Week 4	0.13 ± 0.28	0.28 ± 0.64	NS
Week 7	0.17 ± 0.29	0.16 ± 0.30	NS
Week 10	0.15 ± 0.30	0.28 ± 0.48	NS
Week 13	0.12 ± 0.26	0.20 ± 0.36	NS
*Juice*			
Week 1	0.61 ± 0.50	0.76 ± 0.55	NS
Week 4	0.59 ± 0.57	0.92 ± 0.79	NS
Week 7	0.72 ± 0.47	0.70 ± 0.48	NS
Week 10	0.56 ± 0.48	0.85 ± 0.70	NS
Week 13	0.52 ± 0.47	0.73 ± 0.43	NS
*Milk*			
Week 1	0.28 ± 0.41	0.31 ± 0.38	NS
Week 4	0.32 ± 0.42	0.36 ± 0.42	NS
Week 7	0.30 ± 0.47	0.36 ± 0.39	NS
Week 10	0.21 ± 0.38	0.40 ± 0.43	NS
Week 13	0.30 ± 0.46	0.33 ± 0.42	NS
*Fruit*			
Week 1	0.56 ± 0.58	0.70 ± 0.67	NS
Week 4	0.23 ± 0.43	0.24 ± 0.47	NS
Week 7	0.39 ± 0.42	0.60 ± 0.61	NS
Week 10	0.14 ± 0.36	0.23 ± 0.37	NS
Week 13	0.27 ± 0.33	0.60 ± 0.64	0.027
*Yogurt*			
Week 1	0.21 ± 0.36	0.25 ± 0.40	NS
Week 4	0.21 ± 0.45	0.20 ± 0.34	NS
Week 7	0.24 ± 0.41	0.25 ± 0.42	NS
Week 10	0.22 ± 0.43	0.17 ± 0.34	NS
Week 13	0.16 ± 0.29	0.27 ± 0.39	NS

Table shows mean number of daily servings of each food ± SD. “One serving” is defined as: Eggs = 2 whole eggs; RTE Cereal = 3/4 cup; Hot Cereal = 3/4 cup; Pancakes = 2 small pancakes; Breakfast Meats = 1.5 ounces; Potatoes = 4 ounces; Toast = 2 slices or 1 bagel; Pastries = 2 small; Juice = 8 ounces; Milk = 8 ounces; Fruit = 1/2 cup; Yogurt = 4 ounces. NS, not significant.

Subjects in the EB group consumed an average of one serving of eggs per day at every collection point (5 throughout the study), indicating consistent compliance to the study protocol. This was significantly different from the mean egg consumption frequency for the NEB group. It should be noted that at the first collection, the NEB group consumed an average of 0.56 ± 0.21 servings of eggs that week, which was the result of confusion over group assignments. This was immediately rectified, and from each point onward, the mean daily frequency of egg consumption in the NEB group was <0.1 serving. In order of frequency among all subjects, we found juice to be the most frequently consumed item, while milk and ready to eat cereals were consumed with lower frequency than fruit, potatoes and breakfast meats.

As expected, those in the EB group consumed more average servings of eggs at every time point than those in the NEB group. Other differences were seen only sporadically, starting at week 10, where a difference in mean servings of ready-to-eat cereal and mean servings of toast were apparent. At week 13, differences between the groups were seen in consumed servings of pancakes and fruit. When analyzed by gender (data not shown), the only significant differences seen were in consumption of servings of toast at week 7 (*F* = 0.18 ± 0.32, *M* = 0.39 ± 0.44, *p* = 0.041) and fruit at week 4 (*F* = 0.10 ± 0.23, *M* = 0.29 ± 0.47, *p* = 0.049). When analyzed by final weight change category, significant differences between these groups were seen only sporadically, and these were in the frequency of consumption of meats (weeks 1 and 7) and juice (weeks 4 and 7). We saw no correlation between frequency of consumption of ready to eat cereals and either baseline BMI or weight change among our sample.

Three-day food diaries were collected twice, during the third week of the study (first full week of classes) and again at the final measurement collection session during the last week of the study (last full week of classes), Table 4. However, the proportion of subjects completing food diaries was ~30%. There was no significant difference between groups in mean energy intake at either time point. EB subjects showed a higher mean intake of calories from saturated fat and dietary cholesterol intake at week 3, but these differences were not apparent at week 14. Repeated measures analysis comparing the first and last 3-day food diaries revealed a significant decrease in cholesterol intake in the EB group and a significant increase in cholesterol intake among NEB subjects.

**Table 4:** Average Nutrient Intake (per day) based on 3 day-food diaries.

	*T* = 3 weeks		*T* = 14 weeks	
	Egg Group	Non-Egg Group	Egg Group	Non-Egg Group
	*n* = 20 ^a^	*n* = 17	*n* = 15	*n* = 12
Total Calories	2041 ± 690	1943 ± 969	2347 ± 951	2026 ± 1072
Calories from Carbohydrate	1031 ± 437	1094 ± 545	1278 ± 697	1009 ± 634
Calories from Protein	333 ± 140	305 ± 261	333 ± 144	302 ± 103
Calories from Fat	683 ± 291	585 ± 253	755 ± 452	767 ± 425
Calories from Saturated Fat	285 ± 199	196 ± 114	285 ± 200	213 ± 112
Fiber (g)	15.8 ± 14.5	15.8 ± 10.4	16.2 ± 6.7	15.6 ± 10.9
Cholesterol (mg)	457 ± 187 *	154 ± 116 *	380 ± 157 ^!^	254 ± 179 ^!^
%Cals from Carbohydrate	49.8 ± 7.6 **	55.7 ± 11.8 **	55.1 ± 15.1	48.8 ± 9.0
%Cals from Protein	16.1 ± 3.1	14.7 ± 4.9	14.7 ± 3.6	16.1 ± 5.0
%Cals from Fat	33.9 ± 9.9	31.7 ± 10.4	31.6 ± 12.9	37.7 ± 8.5
%Cals from Saturated Fat	12.4 ± 3.0	10.4 ± 4.4	11.4 ± 5.5	10.9 ± 3.1

^a^ Values are Mean ± SD for the number of subjects indicated. Subjects kept 3-day food diaries at the start and end of the study. Values sharing a common superscript were significantly different from each other: * *p* < 0.05, ** *p* = 0.088 and ^!^ *p* = 0.067.

**Figure 3:**
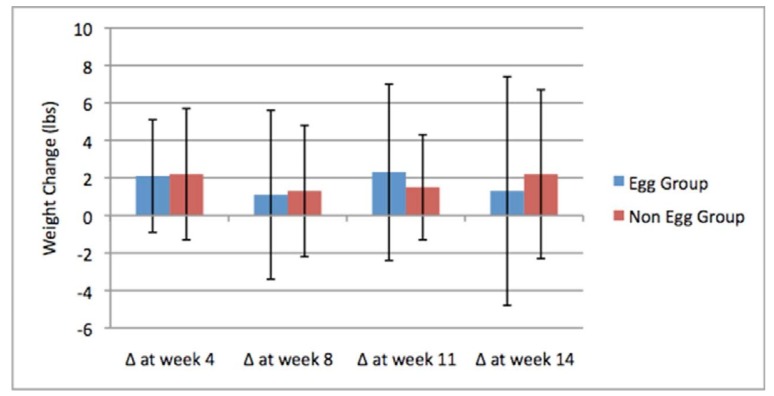
Time course of body weight changes.

With regards to anthropometric measurements, the time-course of the changes in body weight are shown in Figure 3. Among all subjects who completed the final measurement (*n* = 57, 78% of enrolled subjects), a mean weight gain of 1.6 ± 5.3 lbs (0.73 ± 2.41 kg) was observed. Among these subjects, only one lost greater than 5% of their baseline body weight, and six (11%) experienced weight change of greater than 5% of their baseline body weight. Among all subjects completing the final measurement, 28% lost weight (*n* = 16), 53% gained weight (*n* = 30) and 19% (*n* = 11) did not experience a weight change of greater than one pound over the course of the study. There was no significant difference in baseline anthropometric measurement variables between those who lost or gained weight as observed at the final measurement. Significant differences in mean fat mass change were observed between weight change groups at every time point. No significant differences in mean change in fat free mass were observed between weight change groups at any time point until the final measurement. At the final measurement, subjects who had lost weight showed a reduction in fat free mass of 2.2 ± 1.7 lbs (1.00 ± 0.77 kg), while those who gained weight showed a statistically different change of an increase of 0.5 ± 4.2 lbs (0.23 ± 1.91 kg) (*p* = 0.015). Taken together, the results indicate weight change in subjects was primarily due to changes in fat mass. A mixed between-within analysis of variance was conducted to assess the impact of breakfast intervention type on changes in weight and percentage of body fat variables over time. There was a large main effect for time that was significant for weight change (Wilks Lambda = 0.692, *F* = 4.345, *p* < 0.005, partial eta squared = 0.308) and for percent body fat (Wilks Lambda = 0.720, *F* = 3.697, *p* = 0.012, partial eta squared = 0.280). There was no significant interaction effect seen for time and breakfast intervention type on either weight or percentage body fat.

Fasting lipid measurements are shown in Table 5. There were no significant differences between groups in any variables at any time point. A mixed between-within analysis of variance was conducted to assess the impact of breakfast group on changes in fasting plasma cholesterol variables. There was a large main effect for time that was significant for Total Cholesterol (Wilks Lambda = 0.523, *F* = 17.812, *p* < 0.0001, partial eta squared = 0.477), Triglycerides (Wilks Lambda = 0.716, *F* = 5.548, *p* = 0.009, partial eta squared = 0.284) and LDL (Wilks Lambda = 0.667, *F* = 7.000, *p* = 0.003, partial eta squared = 0.333), but not for HDL. There was no significant interaction between intervention type (EB *versus* NEB) and time on any measured parameter.

Values are Mean ± SD. There was no significant difference in body weight change between the two groups at any given time.

**Table 5:** Changes in fasting plasma lipids over time by breakfast group.

	Week 2		Week 8		Week 14	
	EB	NEB	EB	NEB	EB	NEB
Total Cholesterol (mg/dL)	145 ± 22 (*n* = 34)	146 ± 27 (*n* = 28)	152 ± 26 (*n* = 27)	147 ± 26 (*n* = 24)	168 ± 28 (*n* = 27)	164 ± 32 (*n* = 21)
Triglycerides (mg/dL)	83 ± 36 (*n* = 32)	92 ± 38 (*n* = 25)	83 ± 27 (*n* = 23)	91 ± 39 (*n* = 20)	96 ± 41 (*n* = 22)	108 ± 51 (*n* = 18)
LDL Cholesterol (mg/dL)	82 ± 19 (*n* = 30)	83 ± 21 (*n* = 23)	86 ± 22 (*n* = 22)	81 ± 18 (*n* = 20)	97 ± 26 (*n* = 22)	93 ± 23 (*n* = 18)
HDL Cholesterol (mg/dL)	48 ± 12 (*n* = 35)	49 ± 14 (*n* = 32)	52 ± 13 (*n* = 28)	51 ± 14 (*n* = 24)	56 ± 13 (*n* = 28)	54 ± 18 (*n* = 22)
TC/HDLC Ratio	3.2 ± 1.0 (*n* = 33)	3.1 ± 1.0 (*n* = 28)	3.0 ± 0.7 (*n* = 27)	3.1 ± 0.9 (*n* = 24)	3.1 ± 0.8 (*n* = 27)	3.3 ± 1.3 (*n* = 21)

Values are mean ± SD. Measurements were taken at weeks 2, 8 and 14 of the study period. Independent samples *t*-tests revealed no significant differences between study groups in mean TC, LDL, TG and HDL-C concentrations at any time point. A mixed between-within analysis of variance was conducted to assess the impact of breakfast group on changes in fasting plasma cholesterol variables. There was a large main effect for time that was significant for Total Cholesterol (Wilks Lambda = 0.523, *F* = 17.812, *p* < 0.0001, partial eta squared = 0.477), Triglycerides (Wilks Lambda = 0.716, *F* = 5.548, *p* = 0.009, partial eta squared = 0.284) and LDL (Wilks Lambda = 0.667, *F* = 7.000, *p* = 0.003, partial eta squared = 0.333), but not for HDL. There was no significant interaction between intervention type (EB *versus* NEB) and time on any measured fasting plasma cholesterol parameters.

## 4. Discussion

This study aimed to assess differences in weight change between those who consumed eggs regularly at breakfast and those who did not. It was intended to build on previous data that showed eggs to be associated with reduced energy intake and weight loss. In a sample of overweight and obese subjects, an egg breakfast resulted in increased satiety and lower energy intake at lunch as well as at 24 and 36 h compared to an isocaloric bagel breakfast [12]. In a follow-up study [13] conducted over eight weeks, it was shown that the egg breakfast in combination with energy restriction resulted in a 65% greater weight loss compared to an isocaloric bagel breakfast in combination with energy restriction. However weight change was similar for the two breakfast types in subjects that were not consuming calorie-restricted diets. The current study design intended to document the “real life” effects of including eggs with breakfast or not including them. Despite these relatively unrestricted instructions, no differences in energy intake per kilogram body weight were seen between intervention groups.

There is much disagreement in the literature surrounding weight change among incoming college freshmen [21,22,23,24,25,26,27,28,29,30,31,32,33,34,35], with reported studies varying widely in sample sizes, timing of measurements and measurement collection techniques. A significant mean weight change of 1.6 ± 5.3 pounds (*p* < 0.0001) was observed for all subjects in this study who completed the final anthropometric measurement at week 14 of the study. Among studies reporting weight change after only one semester, the mean weight change reported by subjects in this study are similar but comparatively low. Anderson and colleagues reported a mean weight change of 2.86 pounds among a sample of 135 males and females [22]. Other studies reported only changes in body weight among female subjects. Hovell and colleagues reported a mean weight gain of 2.9 pounds among females after one semester [29]. Higher mean weight gains among samples of freshmen women have been reported by Levitsky (4.2 pounds) [30] and Matvienko (3.96 pounds) [31]. Whether the lower weight change noted in our study was attributed to the consumption of breakfast *per se* remains to be established.

Another aim of this study was to document changes in blood lipids and determine any changes that may result from the increase in dietary cholesterol from the EB intervention. Over the last decade, several meta-analyses as well as a highly publicized epidemiology study strongly suggest that daily egg consumption does not adversely affect plasma lipoproteins with regards to the risk for CHD or stroke among healthy individuals [15,36,37]. This may in part be attributed to the fact that in addition to increasing TC and LDL-C, the cholesterol content of eggs also increases HDL-C. Thus for every 100 mg increase in dietary cholesterol, TC increases by 2.2 mg/dL, LDL-C by 1.9 mg/dL and HDL-C by 0.4 mg/dL. Given these values, an additional 1 egg/day would raise TC, LDL-C and HDL-C by 4.7 mg/dL, 4.05 mg/dL and 0.85 mg/dL, respectively. Although, one meta-analysis found that an additional egg consumed daily, increases the TC/HDL-C ratio by 0.04 units, suggesting an increase in CHD risk of 2% [38], the biological and clinical significance of this has been questioned [39]. Thus, in view of the minimal effects that eggs have on plasma lipoproteins and CHD risk, Herron and Fernandez [40] have questioned the appropriateness of dietary guidelines regarding egg consumption.

Herron *et al.* [17], fed normolipidemic men (classified as hypo or hyper-responders to dietary cholesterol) the liquid equivalent of 3 whole eggs per day (~640 mg/day dietary cholesterol) for 4 weeks. Even though total dietary cholesterol intake was ~820 mg/day, no effects on TC, LDL-C, HDL-C or the ratio of LDL-C/HDL-C were noted in the 25 hypo-responders. In the 15 hyper-responders, significant increases in TC and LDL-C were offset by a significant increase in HDL-C, resulting in a significant increase in the LDL-C/HDL-C ratio (1.91 to 2.33). However, these values for the TC/HDL-C ratios were well below the currently recommended value of 4.0. The authors concluded that the additional dietary cholesterol (from 3 eggs/day) did not adversely affect plasma lipoprotein profile, regardless of subjects’ responsiveness to dietary cholesterol. In a study to assess the effects of insulin resistance and obesity on plasma lipoproteins and sensitivity to egg consumption, Knopp *et al.* [41] evaluated 197 healthy subjects who were fed 0, 2 or 4 egg yolks per day for 4 weeks. The extra 850 mg cholesterol (from the 4 eggs) on top of habitual cholesterol intakes of ~300 mg cholesterol/day would have resulted in total cholesterol intakes of ~1150 mg/day. In our study comparing those who consumed eggs for breakfast and those who did not consume eggs for breakfast, there were no differences seen in any plasma lipid variable over the study period.

## 5. Conclusions

Our data show that weight gain, consistent with other reported studies, is modest in college freshmen. Our data, consistent with a growing body of literature, shows that the cholesterol content of eggs does not adversely affect plasma lipids in this free-living population of young, healthy adults.
